# Bacillus Calmette-Guérin–induced trained immunity protects against SARS-CoV-2 challenge in K18-hACE2 mice

**DOI:** 10.1172/jci.insight.157393

**Published:** 2022-06-08

**Authors:** Bao-Zhong Zhang, Huiping Shuai, Hua-Rui Gong, Jing-Chu Hu, Bingpeng Yan, Terrence Tsz-Tai Yuen, Ye-Fan Hu, Chaemin Yoon, Xiao-Lei Wang, Yuxin Hou, Xuansheng Lin, Xiner Huang, Renhao Li, Yee Man Au-Yeung, Wenjun Li, Bingjie Hu, Yue Chai, Ming Yue, Jian-Piao Cai, Guang Sheng Ling, Ivan Fan-Ngai Hung, Kwok-Yung Yuen, Jasper Fuk-Woo Chan, Jian-Dong Huang, Hin Chu

**Affiliations:** 1 CAS Key Laboratory of Quantitative Engineering Biology, Shenzhen Institute of Synthetic Biology, Shenzhen Institutes of Advanced Technology, Chinese Academy of Sciences, Shenzhen, China.; 2 School of Biomedical Sciences, The University of Hong Kong, Pokfulam, Hong Kong Special Administrative Region, China.; 3 State Key Laboratory of Emerging Infectious Diseases, and; 4 Department of Microbiology, Li Ka Shing Faculty of Medicine, The University of Hong Kong, Pokfulam, Hong Kong Special Administrative Region, China.; 5 Centre for Virology, Vaccinology and Therapeutics, Hong Kong Science and Technology Park, Hong Kong Special Administrative Region, China.; 6 Department of Medicine, Li Ka Shing Faculty of Medicine, The University of Hong Kong, Pokfulam, Hong Kong Special Administrative Region, China.; 7 Department of Clinical Microbiology and Infection Control, The University of Hong Kong-Shenzhen Hospital, Shenzhen, Guangdong Province, China.; 8 Guangdong-Hong Kong Joint Laboratory for RNA Medicine, Sun Yat-Sen University, Guangzhou, China.

**Keywords:** COVID-19, Infectious disease, Innate immunity

## Abstract

SARS-CoV-2 has been confirmed in over 450 million confirmed cases since 2019. Although several vaccines have been certified by the WHO and people are being vaccinated on a global scale, it has been reported that multiple SARS-CoV-2 variants can escape neutralization by antibodies, resulting in vaccine breakthrough infections. Bacillus Calmette-Guérin (BCG) is known to induce heterologous protection based on trained immune responses. Here, we investigated whether BCG-induced trained immunity protected against SARS-CoV-2 in the K18-hACE2 mouse model. Our data demonstrate that i.v. BCG (BCG-i.v.) vaccination induces robust trained innate immune responses and provides protection against WT SARS-CoV-2, as well as the B.1.617.1 and B.1.617.2 variants. Further studies suggest that myeloid cell differentiation and activation of the glycolysis pathway are associated with BCG-induced training immunity in K18-hACE2 mice. Overall, our study provides the experimental evidence that establishes a causal relationship between BCG-i.v. vaccination and protection against SARS-CoV-2 challenge.

## Graphical abstract

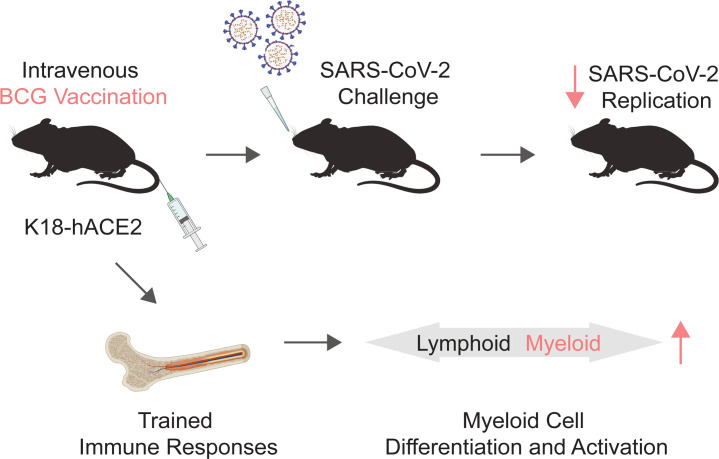

## Introduction

As of March 2022, SARS coronavirus 2 (SARS-CoV-2), the causative agent of Coronavirus Disease 2019 (COVID-19) (1), has infected more than 450 million people and caused over 6.01 million deaths worldwide (2). Data from the preclinical development of SARS-CoV-1 and MERS-CoV candidate vaccines helped to largely eliminate the need for initial exploratory steps of SARS-CoV-2 vaccines, saving considerable time (3). The WHO has so far issued recommendations for the Pfizer-BioNTech, Moderna, AstraZeneca, Janssen COVID, Sinopharm, and SinoVac vaccines (https://www.who.int/emergencies/diseases/novel-coronavirus-2019/covid-19-vaccines/advice). These vaccines have been approved by the WHO for emergency use in different parts of the world. As of November 1, 2021, 7.04 billion doses of vaccine had been administered globally, with 49.4% of the world’s population having received at least 1 dose of a COVID-19 vaccine (4). Importantly, SARS-CoV-2 continues to evolve. To date, the WHO has identified 5 variants of concern (VOCs), including B.1.1.7 (Alpha), B.1.351 (Beta), P.1 (Gamma), B.1.617.2 (Delta), and the most recent B.1.1.529 (Omicron). Accumulating evidence suggests that the VOCs spread more rapidly with potentially altered disease manifestations than the original circulating strain (5–8). It has been reported that multiple SARS-CoV-2 variants can escape neutralization by antibodies, resulting in vaccine breakthrough infections (7, 9–16). Therefore, there is an urgent need for the development of alternative immunization strategies.

The innate immune system is the first line of defense against invading infections (17). It is mediated by a family of pattern recognition receptors (PRRs), which result in the activation of both myeloid (e.g., neutrophils, monocytes, and macrophages) and lymphoid immune cells (e.g., NK and γδ cells), thereby eliminating the majority of pathogens that infect humans on a daily basis (18). Only when this first line of defense is overwhelmed by large numbers of pathogens will the adaptive immune response (B and T cells) be activated. For a long time, it was thought that only the adaptive immune system could mount the immunological memory and defend against recurring infection. This lymphocyte property is the foundation of vaccination against specific infections. However, recent studies showed that innate immune cells can also exhibit adaptive characteristics after certain infections or vaccines, a property that is similar in function to the development of immunological memory, also termed trained immunity (17, 19).

Bacillus Calmette-Guerin (BCG) is a live-attenuated vaccine (LAV) that was developed in the early 20^th^ century to combat tuberculosis (20). It has since become the most widely used vaccine in the world, with approximately 130 million children vaccinated each year. Interestingly, epidemiological studies soon following BCG vaccination’s introduction in Europe in the 1920s found that it significantly reduced infant mortality, which could not be explained solely by a reduction in tuberculosis cases (21). Later, similar studies in other regions, including randomized clinical trials, revealed an up to 50% decrease due to BCG vaccination in young infants (22). Similarly, BCG — but not other childhood vaccinations — was associated with a significantly greater early childhood survival in a high mortality region in West Africa (23). In particular, BCG appears to reduce childhood mortality by protecting against unrelated infectious pathogens, especially respiratory tract infections and new-born sepsis, which are supported by clinical studies (24–27). More recent studies revealed that BCG immunization provides significant protection against viral infections by inducing trained immunity (28–30). In this direction, the potential benefits of BCG vaccination on protecting against COVID-19 have been actively postulated (18, 20, 31–37). Martha et. al analyzed the rate of the day-by-day increase in confirmed COVID-19 cases (134 countries) and deaths (135 countries) in the first 30-day period of country-wise outbreaks, which revealed an association between mandatory BCG policies with a reduction in both COVID-19 cases and fatalities (31). Furthermore, Rivas et al. reported that BCG-vaccinated healthcare staff had lower rates of COVID-19 compared with unvaccinated staff (38, 39). On the basis of these findings, BCG vaccination-induced trained immunity may provide nonspecific protection against SARS-CoV-2 and reduce COVID-19 disease severity, a strategy that may offer important public health benefits.

In this study, we used the K18-hACE2 mouse model to assess whether BCG vaccination offers protection against SARS-CoV-2. We demonstrate that i.v. BCG (BCG-i.v.) vaccination induces a robust innate immune response, limits SARS-CoV-2 replication, and ameliorates SARS-CoV-2–induced weight loss in the K18-hACE2 mouse model. Overall, our study provides the experimental evidence that establishes a causal relationship between BCG-i.v. vaccination and protection against SARS-CoV-2 infection in vivo.

## Results

### BCG-i.v. vaccination offers protection against SARS-CoV-2 in K18-hACE2 mice.

**Figure 1:**
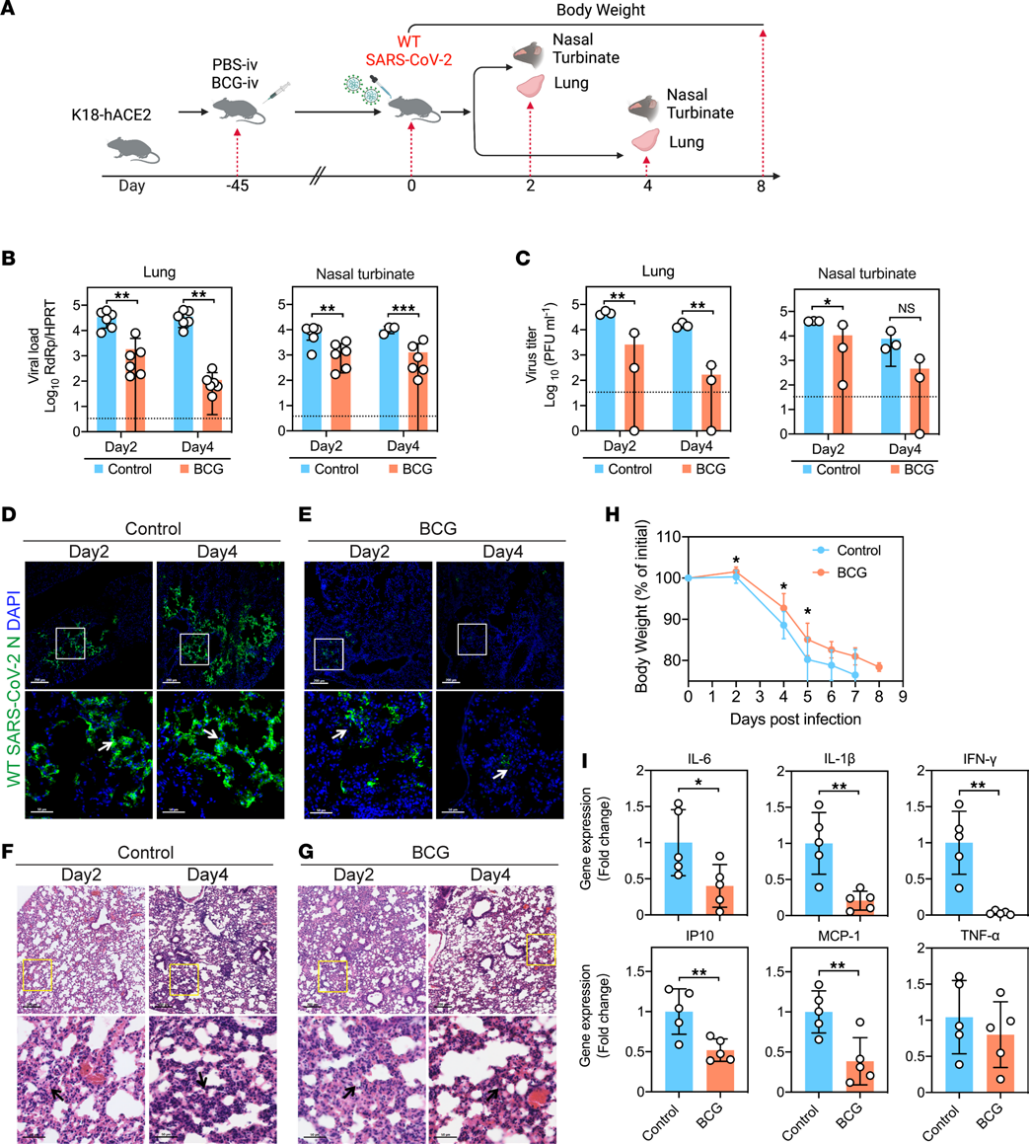
BCG-i.v. immunization protects against WT SARS-CoV-2 in K18-hACE2 mice. (**A**) Schedule of immunization, virus challenge, tissue collection, and BW monitoring. Intranasally inoculated with 1.25 × 10^4^ PFUs of WT SARS-CoV-2 in 20 μL DMEM. (**B**) The viral loads in the lung and nasal turbinate of the K18-hACE2 (*n* = 6) at 2 and 4 days after WT SARS-CoV-2 challenge determined by qPCR. (**C**) The virus titers in the lung and nasal turbinate of the K18-hACE2 (*n* = 3) at 2 and 4 days after WT SARS-CoV-2 challenge determined by plaque assays. (**D** and **E**) Representative images of immunofluorescence staining of the lung tissues of control- or BCG-i.v.–immunized mice at 2 and 4 days after WT SARS-CoV-2 challenge. SARS-CoV-2 was identified using an Ab against SARS-CoV-2 nucleocapsid protein (green signal). Cell nuclei were identified with the DAPI stain (blue signal). The control-immunized mice showed abundant SARS-CoV-2 nucleocapsid protein expression diffusely distributed in the lung (white arrows). The BCG-immunized mice showed markedly less SARS-CoV-2 nucleocapsid protein expression. Scale bar: 200 μm (top) or 50 μm (bottom). (**F** and **G**) Representative images of the H&E-stained lung tissues of control- or BCG-i.v.–immunized mice at 2 and 4 days after WT SARS-CoV-2 challenge. Scale bar: 200 μm (top) or 50 μm (bottom). Peribronchiolar mononuclear cell infiltration (arrow). (**H**) BW changes of WT SARS-CoV-2–infected K18-hACE2 mice with control or BCG-iv vaccination (*n* = 10). Data are shown as mean ± SD. (**I**) The mice were sacrificed at 4 days after virus challenge for lung tissue collection. qPCR analysis of IL-6, IP10, IL-1β, TNF-α, MCP-1, and IFN-γ mRNA expression level (*n* = 5). Data are shown as mean ± SD. Statistical significance was calculated using unpaired 2-tailed Student’s *t* test. *P < 0.05, **P < 0.01, ***P < 0.001. Data from 3 independent experiments were shown. Dotted line represents detection limits.

BCG vaccination has been shown to provide nonspecific protection in multiple models of virus infection (29, 33). To evaluate the potential of BCG vaccine against SARS-CoV-2 infection, we injected K18-hACE2 mice i.v. with BCG vaccine and i.n. challenged the mice with SARS-CoV-2 WT 45 days after vaccination and collected tissues for virological and histological assessment 2 and 4 days post infection (dpi) (Figure 1A). As shown in Figure 1B, viral gene copies in lung tissue of BCG-i.v. vaccinated mice were significantly lower than that of the control nonimmunized mice at 2 dpi (*P* ≤ 0.01) and 4 dpi (*P* ≤ 0.01) by 1.5 to 3.5 log, respectively. In addition, BCG-i.v.–immunized K18-hACE2 mice were similarly protected against SARS-CoV-2 infection in the upper respiratory tract (nasal turbinate) (Figure 1B). Consistently, significant suppression of infectious SARS-CoV-2 viral particles in the lung (2 dpi: *P* ≤ 0.01; 4 dpi: *P* ≤ 0.01) and nasal turbinate (2 dpi: *P* ≤ 0.05; 4 dpi: *P* > 0.05) were confirmed in BCG-i.v.–administered mice (Figure 1C).

In line with findings on viral load and virus titer, immunofluorescence staining assay detected abundant SARS-CoV-2 nucleocapsid protein expression diffusely distributed in the lung of control mice (Figure 1D), which was markedly reduced among the BCG-i.v.–immunized mice (Figure 1E). In particular, SARS-CoV-2 nucleocapsid protein is only marginally detected from the lung of BCG-i.v.–vaccinated mice at 4 dpi, suggesting robust inhibition of virus replication in the lung upon BCG-i.v. vaccination (Figure 1E). Interestingly, on examination of the histopathological changes, we noticed that BCG-i.v. vaccination modestly increased the level of mononuclear inflammatory cell infiltration in the lung (Figure 1, F and G). In a parallel set of experiments, our results indicated that BCG-i.v. vaccination alone resulted in a mild degree of inflammatory cell infiltration in the lung at 45 days following vaccination, and modestly upregulated the baseline expression of a number of cytokines in the lung (Supplemental Figure 1; supplemental material available online with this article; undefinedDS1). Nevertheless, BCG-i.v. vaccination significantly reduced the BW loss from day 2 to day 5 after SARS-CoV-2 infection compared with the control mice, suggesting an overall beneficial role of BCG-i.v. vaccination on SARS-CoV-2 infection in the K18-hACE2 mice (Figure 1H). Importantly, despite the upregulated baseline, the expression of most of these cytokines (IL-6, IP10, IL-1β, MCP-1, and IFN-γ) were significantly lower in the BCG-i.v.–immunized mice upon SARS-CoV-2 infection. In particular, IL-6, a representative proinflammatory cytokine that is implicated in SARS-CoV-2 pathogenesis, was 2.4-fold (*P* ≤ 0.05) lower than that of the control mice at 4 dpi (Figure 1I).

**Figure 2:**
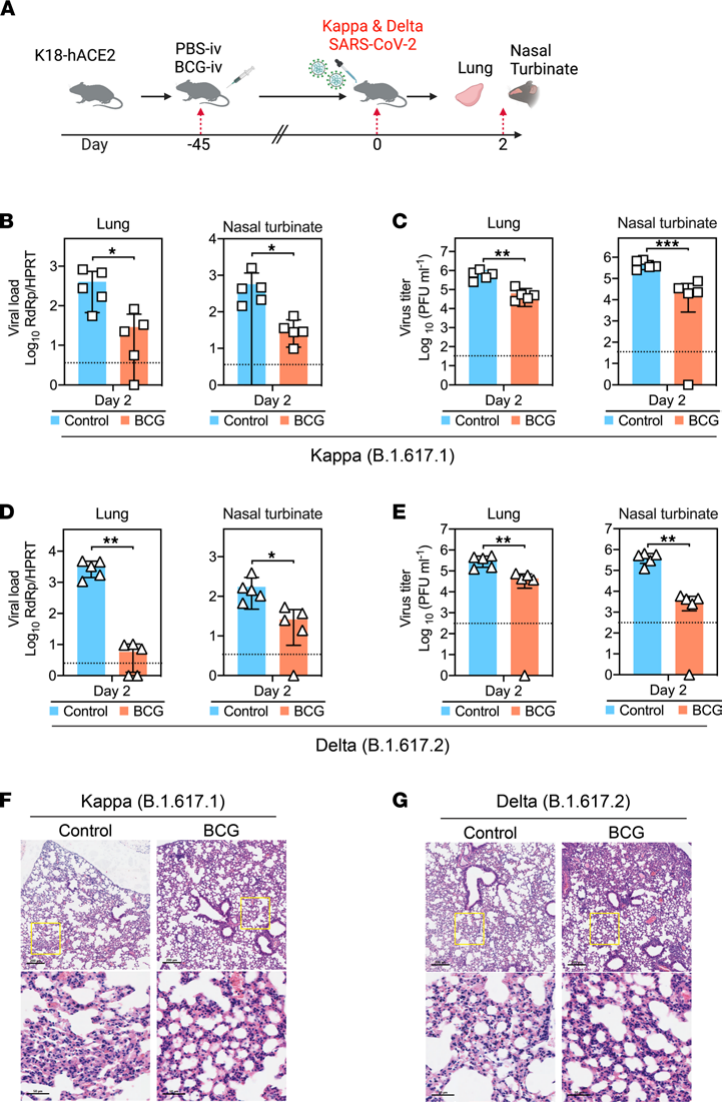
BCG-i.v. immunization protects against SARS-CoV-2 variants in K18-hACE2 mice. (**A**) Schedule of immunization, virus challenge, tissue collection, and BW monitoring. After 45 days of BCG training, K18-hACE2 mice were i.n. inoculated with 1.25 x 10^4^ PFUs of SARS-CoV-2 variants in 20 μL DMEM. The mice were sacrificed at 2 days after virus challenge for tissue collection. (**B**) Viral loads in the lung and nasal turbinate of the K18-hACE2 mice (*n* = 5) at 2 days after SARS-CoV-2 Kappa (B.1.167.1) challenge determined by RT-qPCR. (**C**) Virus titers in the lung and nasal turbinate of the K18-hACE2 mice (*n* = 5) at 2 days after SARS-CoV-2 Kappa (B.1.167.1) challenge determined by plaque assays. (**D**) Viral loads in the lung and nasal turbinate of the K18-hACE2 mice (*n* = 5) at 2 days after SARS-CoV-2 Delta (B.1.167.2) challenge determined by RT-qPCR. (**E**) Virus titers in the lung and nasal turbinate of the K18-hACE2 mice (*n* = 5) at 2 days after SARS-CoV-2 Delta (B.1.167.2) challenge determined by plaque assays. (**F** and **G**) Representative images of the H&E-stained lung tissues of control- or BCG-i.v.–immunized mice at 2 days after SARS-CoV-2 Kappa or Delta challenge. Scale bar: 200 μm or 50 μm. Data are presented as mean ± SD. Statistical significance was calculated using unpaired 2-tailed Student’s *t* test (*P < 0.05, **P < 0.01, ***P < 0.001). Data from 3 independent experiments were shown. Dotted line represents detection limits.

The emergence of SARS-CoV-2 variants with potential escape mutations is a major concern (7). We next evaluated the potential protection efficacy of BCG-i.v. vaccination against emerging SARS-CoV-2 variants B.1.617.1 (Kappa) and B.1.617.2 (Delta) (Figure 2A). As demonstrated in Figure 2B, B.1.617.1 viral gene copies in lung tissue and nasal turbinate of BCG-i.v.–vaccinated mice were 14.4-fold (*P* ≤ 0.05) and 16-fold (*P* ≤ 0.05) lower than that of the control-immunized mice at 2 dpi, respectively. In agreement with these findings, the virus titers in lung tissue (*P* ≤ 0.01) and nasal turbinate (*P* ≤ 0.001) of BCG-i.v.–vaccinated mice were both significantly lower than that of the control-immunized mice (Figure 2C). Similarly, BCG protects against B.1.617.2 infection as evidenced by the significantly reduced virus replication in the lung and nasal turbinate (Figure 2D). The viral RNA copies in the lungs and nasal turbinate of BCG-i.v.–vaccinated mice were 418-fold (*P* ≤ 0.01) and 5.4-fold (*P* ≤ 0.05) lower than that of the control-immunized mice, respectively (Figure 2D). Consistent with the viral load results, the infectious virus titer in lung tissue (*P* ≤ 0.01) and nasal turbinate (*P* ≤ 0.01) of BCG-i.v.–vaccinated mice were significantly lower than that of the control-immunized mice (Figure 2E). Interestingly, similar to SARS-CoV-2 WT, the extent of mononuclear inflammatory cell infiltration in the lung of B.1.617.1- or B.1.617.2-infected mice was modestly increased by prior BCG vaccination (Figure 2, F and G). In addition to BCG-i.v. vaccination, we also evaluated the protection efficacy of s.c. BCG (BCG-s.c.) vaccination against SARS-CoV-2 WT, Kappa, and Delta. As demonstrated in Supplemental Figure 2, s.c. immunization with BCG did not offer protection against subsequent challenges from SARS-CoV-2 WT, Kappa (B.1.617.1), or Delta (B.1.617.2) (Supplemental Figure 2).

Taken together, our results suggest that BCG-i.v. vaccination in mice protects against the infection of SARS-CoV-2 WT and variants, which is evidenced by the reduction in the generation of infectious viral progeny from the lung and nasal turbinate, the markedly reduced viral antigen expression in the lung, the lowered proinflammatory response upon virus infection, and the significantly improved BW loss in the infected animals.

### Administration of BCG-i.v. triggers hematopoietic stem cell expansion.

**Figure 3:**
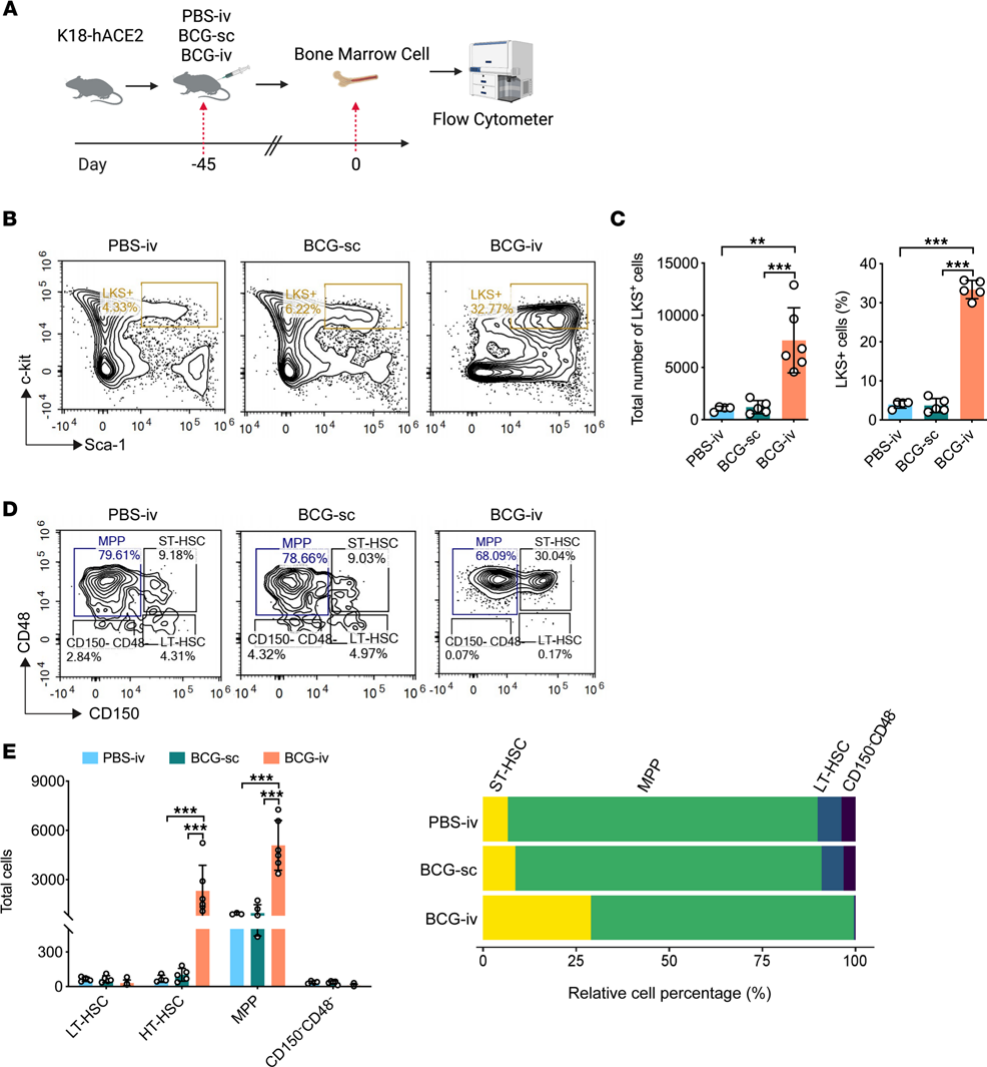
Administration of BCG-i.v. triggers expansion of LKS^+^ BM cells. (**A**) Schematic representation of the experimental procedure for Flow cytometry. K18-hACE2 mice were i.v. or s.c. inoculated with BCG. Mice in control group were injected i.v. with PBS. Mice BM cells were collected on day 45 after BCG vaccination or PBS treatment and processed to flow cytometry. (**B**) Representative FACS blots of BM-LKS^+^ cells in nonvaccinated (PBS-i.v.), BCG-s.c.–, or BCG-i.v.–vaccinated mice (left to right). (**C**) The total cell number (right panel) and relative cell percentage (left panel) of BM-LKS^+^ cells in nonvaccinated (PBS-i.v.), BCG-s.c.–, or BCG-i.v.–vaccinated mice. Data are presented as mean ± SD. Statistical significance was calculated using 1-way ANOVA test (*P < 0.05, **P < 0.01, ***P < 0.001). (**D**) Representative FACS blots of LT-HSCs, ST-HSCs, and MPPs in nonvaccinated (PBS-i.v.), BCG-s.c.–, or BCG-i.v.–vaccinated mice (left to right). (**E**) The quantification of the total cell numbers (right panel) and relative cell percentage (left panel) of LT-HSCs, ST-HSCs, and MPPs in nonvaccinated (PBS-i.v.), BCG-s.c.,– or BCG-i.v.–vaccinated mice. Statistical significance was calculated using 2-way ANOVA test (**P < 0.01, ***P < 0.001).

To examine the potential mechanisms underlying the observation that BCG-i.v. vaccination confers protection against SARS-CoV-2, we immunized K18-hACE2 mice i.v. or s.c. with the BCG Tokyo 172 strain (Figure 3A). To classify hematopoietic stem cells (HSCs) and progenitor cells, mouse BM cells were collected on day 45 after BCG vaccination or PBS treatment and processed to flow cytometry analysis. Consistent with a previous report (40), we found that the BM-LKS^+^ population was significantly upregulated in BCG-i.v.–vaccinated mice compared with nonvaccinated (PBS-i.v.) or BCG-s.c.–vaccinated mice, both in percentage and in the total number of cells (Figure 3, B and C). We next specifically evaluated the ratios of multipotent progenitors (MPPs; LKS^+^CD150^–^CD48^+^), long-term HSCs (LT-HSCs; LKS^+^CD150^+^CD48^–^), and short-term HSCs (ST-HSCs; LKS^+^CD150^+^CD48^+^) (Figure 3, D and E). In comparison to nonvaccinated (PBS-i.v.) mice or BCG-s.c.–vaccinated mice, the number of cells and the percentage of ST-HSCs and MPPs were both considerably increased in BCG-i.v.–vaccinated mice (Figure 3E). These findings suggest that BCG vaccination triggers BM HSC growth and boosts the expansion and differentiation of HSC.

### Administration of BCG-i.v. enhances myelopoiesis in the BM.

**Figure 4:**
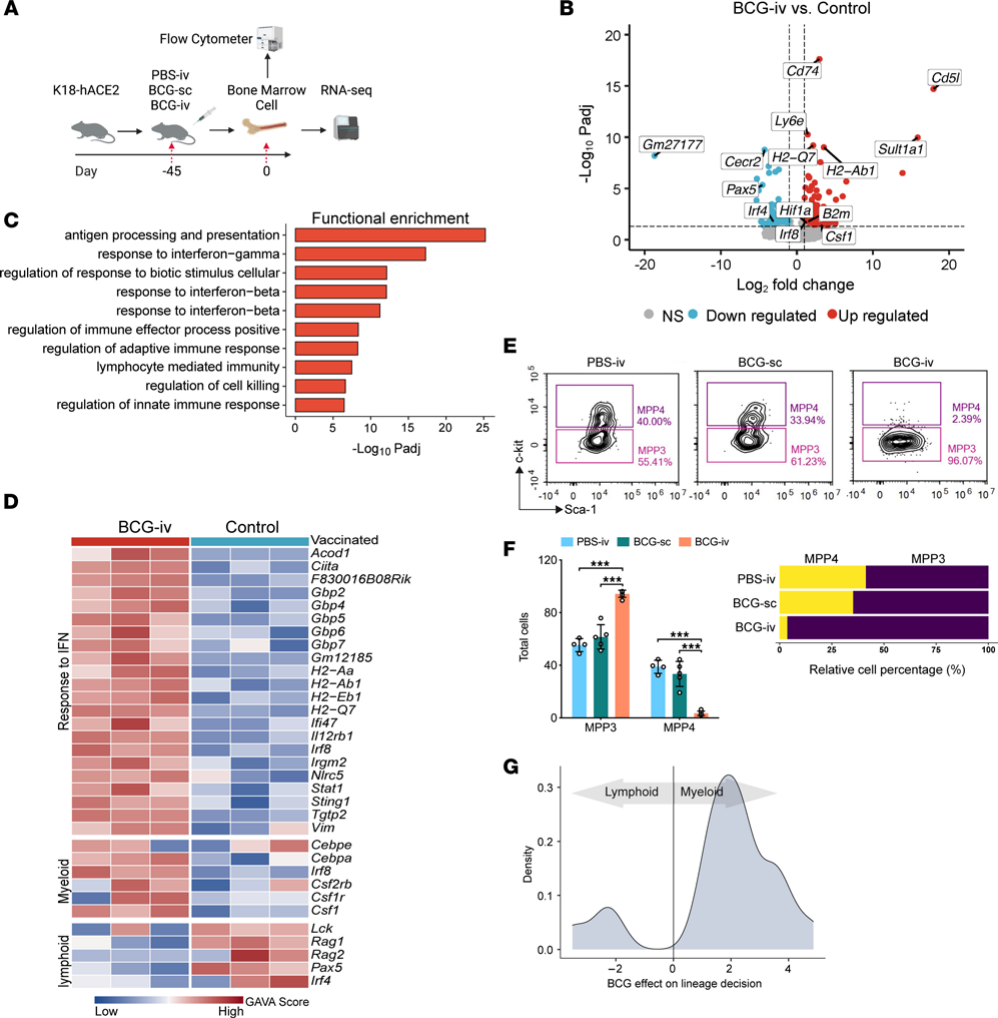
BCG-i.v. vaccination promotes myelopoiesis in BM cells. (**A**) Schematic representation of the experimental procedure for RNA-seq or flow cytometry. K18-hACE2 mice were i.v.- or s.c.-inoculated with BCG. Mice in the control group were injected i.v. with PBS. Mice BM cells were collected on day 45 after BCG vaccination or PBS treatment and processed to RNA-seq or flow cytometry. (**B**) The profile of differentially expressed genes comparing BM cells from BCG-i.v.–vaccinated and control-vaccinated mice. (**C**) Top 10 most strongly enriched GO biology process terms among upregulated genes in BCG-i.v.–immunized mice. (**D**) Gene expression levels of IFN response genes and markers of myeloid and lymphoid cells. (**E**) Representative FACS blots of the frequencies of MPP3 and MPP4 populations in nonvaccinated (PBS-i.v.), BCG-s.c.–, or BCG-i.v.–vaccinated mice (left to right). (**F**) The quantification of the total cell numbers (left panel) and relative cell percentage (right panel) of MPP3 and MPP4 (among all MPPs) in nonvaccinated (PBS-i.v.), BCG-s.c.–, or BCG-i.v.–vaccinated mice. Data are presented as mean ± SD. Statistical significance was calculated using 2-way ANOVA test (***P < 0.001). (**G**) Impact of BCG-i.v. vaccination on the polarization of BM cells toward the myeloid lineage.

To further investigate the mechanism of BCG-induced protection against SARS-CoV-2 infection, we performed bulk RNA-seq on BM cells collected from BCG-vaccinated and control K18-hACE2 mice (Figure 4A). Differential expression analysis revealed 106 genes that were significantly upregulated upon BCG vaccination (Figure 4B). Further analysis revealed that these differentially expressed genes were highly enriched in pathways on the regulation of both innate and adaptive immune responses (Figure 4C). Consistent with previous studies (40, 41), genes relate to antigen processing and presentation, response to type I and II IFN, and myeloid cell differentiation were significantly upregulated after BCG vaccination (Figure 4C). In addition, our results suggested that genes involved in myeloid-lineage maturation (e.g., *Cebpe, Cebpa,* and IFN regulatory factor 8 [*Irf8*]), as well as key cytokines for the proliferation of macrophages and monocytes (e.g., *Csf1*) were similarly upregulated in BM cells after BCG vaccination (Figure 4D). In contrast, lymphoid transcription factors (TFs), *Irf4* and *Pax5,* were downregulated (Figure 4D).

To confirm the transcriptome results, we next employed flow cytometry to determine the number of MPP3s (LKS^+^CD150^–^CD48^+^CD34^+^Flt3^–^) and MPP4s (LKS^+^CD150^–^CD48^+^CD34^+^Flt3^+^) in nonvaccinated (PBS-i.v.), BCG-s.c.–vaccinated, or BCG-i.v.–vaccinated mice, and demonstrated a markedly increased proportion of MPP3s, but not of MPP4s, in BCG-i.v.–vaccinated mice, both in the number and percentage of cells (Figure 4, E and F). However, nonvaccinated (PBS-i.v.) treated mice and BCG-s.c.–vaccinated mice did not show a significant difference in these populations. These results demonstrated that BCG-i.v. vaccination promotes BM-LKS^+^ cell growth and the polarization of MPPs toward the myeloid lineage, which indicated cells in BM were driven to polarize toward the myeloid lineage instead of the lymphoid lineage upon BCG vaccination (Wilcoxon’s rank-sum test *P* < 0.01) (Figure 4G). These findings are consistent with results from a previous study (40). Collectively, with transcriptomic profiling and flow cytometry analysis, our results suggest that BCG-i.v. vaccination in the K18-hACE2 mice triggers upregulation of the innate immune response and the polarization of cells in BM toward the myeloid lineage.

### BCG-trained immunity enhanced protection against SARS-CoV-2.

**Figure 5:**
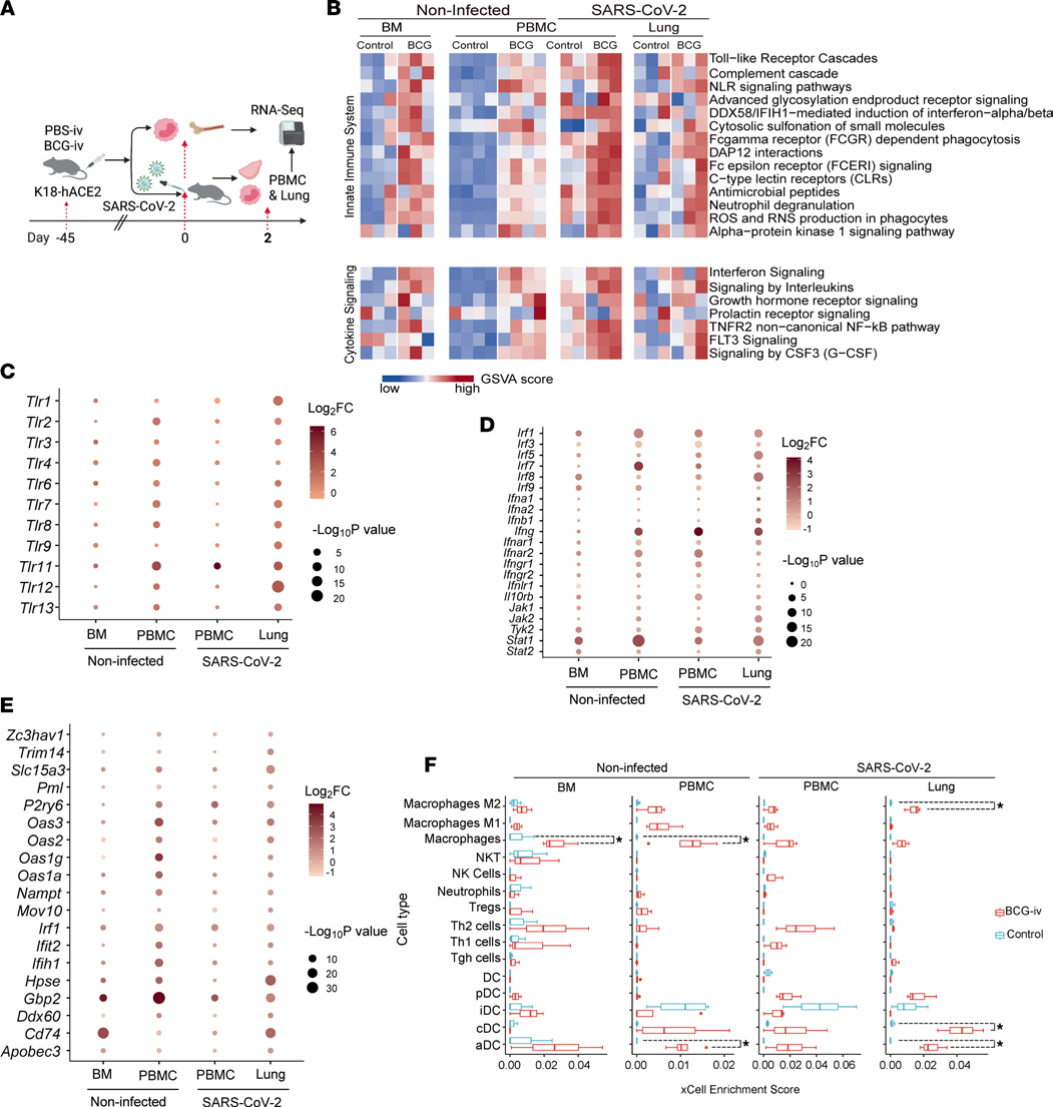
BCG-i.v. vaccination enhances immune responses upon SARS-CoV-2 infection. (**A**) Schematic representation of the RNA-seq experiment design. Tissues were collected from either BCG-i.v.–vaccinated or control-vaccinated mice. (**B**) GSVA analysis on immune-related pathways, GSVA scores were normalized between samples that came from the same tissue, and a higher score indicated higher gene expression of genes from the same gene sets compared with lower score samples. (**C**) Expression changes of genes in the toll-like receptor family. (**D**) Expression changes of genes that regulate IFN signaling, and (**E**) Expression change of antiviral IFN-stimulated genes. Dot plot depicting expression change of innate immunol protection-related genes comparing BCG vaccination to control, dot size, and transparency denotes *P* value and fold change calculated using DESeq. (**F**) Cell type enrichment estimation by xCell. Statistical differences were determined with unpaired 2-tailed Student’s *t* test (*P < 0.05).

Next, we performed RNA-seq on both PBMCs and lung tissues collected from BCG-i.v.–vaccinated and unvaccinated control mice with or without SARS-CoV-2 challenge (Figure 5A). To translate the transcriptomic profiles of these samples to functional data, we carried out Gene Set Variation Analysis (GSVA) utilizing the immune system pathways recorded in the Reactome database (42). Our results indicated that the host innate immune response pathways were activated by BCG vaccination, which were further triggered by SARS-CoV-2 infection (Figure 5B). As previously reported (42), TLR2 and TLR4 serve as key PRRs following BCG inoculation. Our data showed that these 2 TLRs were significantly upregulated in PBMCs upon BCG-i.v. vaccination (Figure 5C). Compared with unvaccinated mice, in mice with prior BCG-i.v. vaccination, the expression of almost all TLRs was upregulated in the lung upon SARS-CoV-2 infection, including *Tlr7* that recognizes single-stranded RNA and *Tlr3* that recognizes double-stranded RNA in endosomes (Figure 5C).

In agreement with these findings, BCG-i.v. vaccination enhanced the expression of TFs including *Irf5*, a central mediator of TLR7 signaling, as well as *Irf8* and *Stat1/2*, which regulate IFN expression in the lung upon SARS-CoV-2 infection (Figure 5D). These upregulated TFs eventually led to the significantly increased expression of antiviral IFNs such as IFN-β (*Ifnb1*) (Figure 5D) and subsequently the expression of a large repertoire of IFN-stimulated genes (ISGs) (Figure 5E). Furthermore, we analyzed the enrichment of 30 immune cell types among our samples from gene expression profiles. In brief, enrichment single-sample gene set enrichment analysis (ssGSEA) scores were calculated with default signature genes from xCell. The scores were compared between samples that were extracted from the same tissue. Our results suggested that macrophages and DCs were significantly enriched after BCG-i.v. vaccination (Figure 5F), indicating a crucial role of myeloid cells in BCG-induced trained immunity in protecting against SARS-CoV-2 infection in the K18-hACE2 mice.

### Glycometabolism reprogramming of BCG-trained immunity.

**Figure 6:**
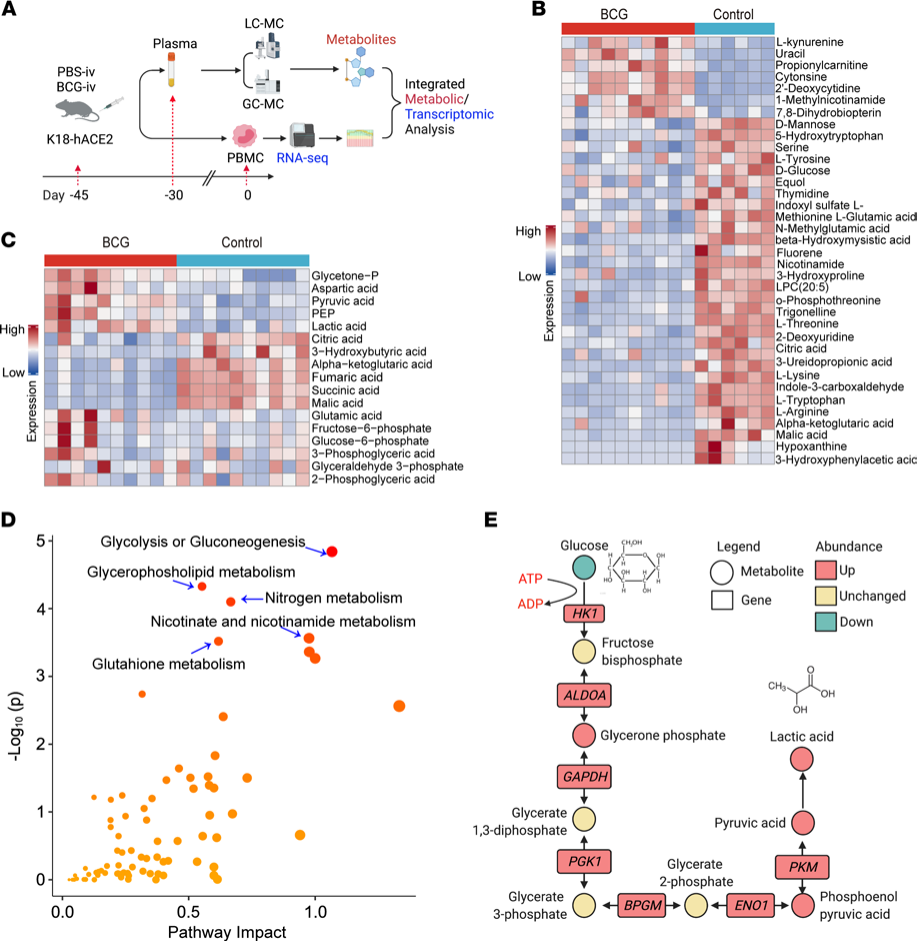
Integrative analysis of metabolomics and transcriptomics based on BCG-i.v.–vaccinated mouse plasma. (**A**) Schematic representation of plasma-based metabolomics and transcriptomics. (**B**) Heatmap of 37 significantly changed metabolites based on LC-MS–untargeted metabolomics. Each bar represented a metabolite colored by its average intensity on a normalized scale from blue (decreased level) to red (increased level). (**C**) Heatmap of 17 quantified metabolites based on GC-MS–targeted metabolomics. Each bar represented a metabolite colored by its average intensity on a normalized scale from blue (decreased level) to red (increased level). (**D**) The integrated metabolic/transcriptomics pathway analysis was constructed by Metaboanalyst. The *y* axis, “-log_10_(p)”, represented the transformation of the original *P* value calculated from the enrichment analysis. The *x* axis, “Pathway Impact”, represented the value calculated from the pathway topology analysis. The integration method was chosen as combining queries in which genes and metabolites are pooled into a single query and used to perform enrichment analysis. (**E**) The glycolysis/gluconeogenesis pathway was constructed based on all significantly changed metabolites and genes mapped along this pathway.

BCG induction of trained immunity in myeloid cells is characterized by a shift of the glucose metabolism toward glycolysis, which is critical for the induction of the epigenetic reprogramming and functional changes underlying BCG-induced trained immunity (43). To evaluate the metabolic changes in BCG-i.v.–vaccinated K18-ACE2 mice, we harvested mouse plasma and PBMCs from BCG-i.v.–vaccinated K18-ACE2 mice at 15 and 45 days following BCG vaccination, respectively (Figure 6A). The mouse plasma was evaluated with liquid chromatography/gas chromatography-mass spectrometry–based (LC/GC-MS–based metabolomic analysis and the transcriptomic profiles of PBMCs were analyzed with RNA-seq (Figure 6A). As shown in Figure 6B, LC-MS–based untargeted metabolomics detected a total of 265 metabolic features in positive mode and 407 metabolic features in negative mode. A total of 37 metabolites were finalized (including 14 standard-confirmed metabolites) and their abundance was visualized by heatmap (Figure 6B and Supplemental Table 1). Among them, a total of 30 metabolites were significantly downregulated and 7 metabolites were upregulated after BCG-i.v. vaccination. Interestingly, glucose and TCA cycle intermediates, including alpha-ketoglutaric acid, citric acid, malic acid, and glutamic acid, were downregulated after BCG-i.v. vaccination (Figure 6B). To further dissect the changes in glycometabolism, GC-MS–based targeted metabolomics were applied to investigate the critical metabolites of glycolysis and TCA cycle pathways. As demonstrated in Figure 6C, a total of 11 metabolites (all standard confirmed) were significantly changed in BCG-vaccinated mouse plasma, with 5 metabolites increased and 6 metabolites decreased in amount (nmol/sample) upon BCG-i.v. vaccination (Figure 6C and Supplemental Table 1). Intriguingly, among these 11 significantly altered metabolites, 5 decreased metabolites including citric acid, alpha-ketoglutaric acid, malic acid, fumaric acid, and succinic acid mapped to the TCA cycle, while 4 increased metabolites, including glycetone phosphate, pyruvic acid, phosphoenolpyruvic acid, and lactic acid mapped to the glycolysis pathway (Figure 6C). These results reveal that BCG-i.v. vaccination in the K18-ACE2 mice activates the glycolysis pathway, which may contribute to the observed BCG-induced trained immunity in our K18-ACE2 model against SARS-CoV-2 infection (43).

Next, we performed integrated transcriptomic-metabolomic analysis by the MetaboAnalyst tool (https://www.metaboanalyst.ca/) to investigate the most highly activated metabolic pathways upon BCG-i.v. vaccination (44, 45). Our results suggested that the glycolysis pathway remained as the most activated pathway based on integrative pathway analysis (Figure 6D), which was due to the abundantly upregulated metabolites and genes along this pathway upon BCG vaccination (Figure 6E). In particular, the decreased glucose level was associated with an increased glucose metabolism that generated an increased level of pyruvic acid, which was converted to lactic acid (for glycolysis) instead of citric acid (for the TCA cycle), a phenomenon similar to the Warburg Effect (46) (Figure 6E). Altogether, these findings suggest that BCG vaccination in K18-ACE2 mice activates glycolysis, which is critical for BCG-induced trained immunity (43) that may offer protection to the infection of SARS-CoV-2 and SARS-CoV-2 variants in K18-ACE2 mice.

## Discussion

BCG is a live attenuated strain generated from a Mycobacterium bovis isolate and is one of the most frequently applied vaccinations in the world (20). Despite its original purpose as a tuberculosis vaccine, numerous studies have demonstrated its potential to provide effective protection against off-target infections (34, 47). In particular, the BCG vaccination is known to provide protection against respiratory tract infections by inducing trained immunity (17, 19, 28, 43). Based on this existing knowledge, BCG has been postulated as providing protection against COVID-19 (18, 31, 33, 34, 36–38). In the current study, we demonstrate that the BCG-i.v. vaccination induces a robust innate immune response in K18-hACE2 mice, limits the replication of SARS-CoV-2 WT and Kappa/Delta variants in both nasal turbinate and the lung, lowers the virus-induced proinflammatory response, and ameliorates SARS-CoV-2–induced weight loss in the K18-hACE2 mouse model. Overall, our study provides key experimental evidence that establishes a causal relationship between BCG-i.v. vaccination and protection against the SARS-CoV-2 infection in vivo.

To evaluate the underlying mechanism behind the BCG-i.v. vaccination-induced protection against SARS-CoV-2 in the K18-hACE2 mouse model, we performed RNA-seq on samples extracted from infected mice. Our data revealed that BCG-i.v. vaccination resulted in upregulation of the innate immune response, which is in line with previous reports (29, 40, 43). Upon SARS-CoV-2 infection, PRRs (e.g., *Tlr3* and *Tlr7*) and TFs (e.g., *Irf5, Irf8,* and *Stat1/2*) in BCG-i.v.–vaccinated mice are further upregulated in comparison to the control-vaccinated mice, resulting in the significantly increased expression of antiviral IFNs and IFN-stimulated genes. These enhanced antiviral responses significantly reduced virus replication in the nasal turbinate and the lung of the infected animals. These findings are consistent with a recent report (48) that was published while the current study was under peer review. Importantly, BCG-i.v. vaccination resulted in significantly lower levels of evaluated proinflammatory cytokines, including IL-6, IP10, IL-1β, MCP-1, and IFN-γ in the lungs of infected mice at day 4 after infection (Figure 1I), presumably due to the substantially reduced virus replication. This finding further highlighted the potential of the BCG vaccination in protecting against SARS-CoV-2 since the level of these proinflammatory cytokines, such as IL-6 and IP10, are positively associated with an unfavorable prognosis and worse outcomes in COVID-19 (49, 50). Overall, our in vivo data from the K18-ACE2 mouse model are congruous with the current knowledge that vaccination with BCG results in activation of antiviral mechanisms that leads to decreased virus replication and, thus, lower local/systemic inflammation with milder diseases (17, 18).

Previous studies suggest that epigenetic reprogramming of cells from the myeloid lineage can result in the increased trained immunity upon BCG vaccination (29, 30), which is characterized by a shift of the glucose metabolism toward glycolysis (43). Our data revealed that BCG-i.v. vaccination in K18-hACE2 mice resulted in the polarization of cells toward the myeloid lineage, consistent with a recent report (40). Through metabolomics analyses, our results revealed that a high rate of glycolysis was sustained while the TCA cycle was suppressed. In particular, our data revealed that pyruvic acid, which is a critical intermediate bridging glycolysis of the TCA cycle, is preferentially converted to lactic acid instead of critic acid of the TCA cycle. This is consistent with the accumulating body of evidence that indicates that glycolysis is upregulated in BCG-induced trained immunity and that glycolysis with increased lactate production is a core hallmark of trained immunity (45, 51). However, our current study did not provide direct evidence that these metabolite levels mirror metabolic changes in immune cells, and further studies are needed to confirm the causal relationship between metabolic changes and BCG-induced protection in the context of SARS-CoV-2 infection.

Overall, our study provides evidence that demonstrates BCG-i.v. vaccination protects against SARS-CoV-2 challenge in vivo. Importantly, despite BCG vaccination not preventing the K18-hACE2 mice from SARS-CoV-2 infection, it significantly attenuates virus replication, reduces the excessive proinflammatory responses, and ameliorates SARS-CoV-2–induced BW loss in the infected mice. These findings suggest that BCG vaccination in humans may not prevent infection itself but may reduce severe outcome of the infection. Overall, our findings provide support for the current hypothesis that BCG vaccination can protect against COVID-19 (18, 20, 31–34). It is important to note that our study suggested that BCG-i.v. vaccination offered protection against SARS-CoV-2 challenge. However, current BCG vaccination for tuberculosis prevention is administered through the intradermal route but not the i.v. route. Therefore, further clinical trials are needed to evaluate whether a clinically acceptable BCG vaccination strategy can help to combat the current COVID-19 pandemic.

## Methods

### BCG and animal.

An attenuated live Bacillus Calmette-Guérin (BCG, Tokyo 172 strain) was purchased from the Japan BCG Lab. Heterogenous K18-hACE2 C57BL/6J mice, 2B6.Cg-Tg(K18-ACE2)2Prlmn/J, were obtained from The Jackson Laboratory.

### Vaccination of K18-hACE2 C57BL/6J mice.

K18-hACE2 C57BL/6J mice were i.v. or s.c. with the BCG Tokyo 172 strain (~1 × 10^6^) of single-suspended BCG in 100 μL of 0.9% solution of sodium chloride as previously described (40).

### Virus.

SARS-CoV-2 WT virus HKU-001a (GenBank accession number MT230904) was an archived clinical isolate as previously described (52). SARS-CoV-2 B.1.617.1 or B.1.617.2 were isolated from the nasopharyngeal aspirates of patients with laboratory-confirmed COVID-19 in Hong Kong (53). All viruses were propagated and titrated in Vero-TMPRSS2 cells with plaque assay as we previously described (54). In vitro and in vivo experiments involving infectious WT SARS-CoV-2, B.1.617.1 or B.1.617.2, were performed in a biosafety level 3 laboratory and strictly followed the approved standard operation procedures (53).

### In vivo virus challenge.

For virus challenge in mice, K18-hACE2 transgenic mice were anesthetized with ketamine and xylazine, followed by intranasal inoculation with 20 μL/mouse of WT or B.1.617.1 or B.1.617.2 SARS-CoV-2 at 1.25 × 10^4^ PFU/mouse as we previously described (53). On days 2 and 4 after virus challenge, the animals were euthanized for harvesting organs for viral load titration and histology staining.

### RNA extraction and quantitative PCR (qPCR).

PBMCs were isolated from mice as we previously described (55). Briefly, the blood (1:1 diluted with sterile PBS) was gently layered on the top of Histopaque (Sigma-Aldrich, 10831) and centrifuged at 400*g* for 30 minutes at room temperature in a swing out bucket. In the separation phase, PBMCs were aspirated and washed twice with PBS. After washing, the cells were resuspended in 1 mL of RBC lysing buffer (BioLegend, RBC Lysis Buffer) and incubated for 5 minutes at room temperature, after which the lysis reaction was stopped with 3 mL of PBS. Following 2 washes with PBS, the cell pellet was stocked at –80°C until further use. BM cells extracted from mice femur were washed with PBS, then centrifuged at 550*g* for 5 minutes, and the cell pellet was stocked at –80°C until further use (55). The harvested lung and nasal turbinate were homogenized in DMEM with Tissue Lyzer II, followed by centrifugation at 10,000*g* for 5 minutes. The lysates were lysed with RLT buffer (Qiagen, 79216) at room temperature for 10 minutes followed by RNA extraction. The host gene expression was quantified using the Qiagen QuantiNova SYBR Green RT-PCR kit (208154). Primer lists were included as Supplemental Table 2. Viral gene copies were quantified with the Qiagen QuantiNova Probe RT-PCR kit (208354) using sequence-specific probes and primers targeting the RNA-dependent RNA polymerase (Forward: 5′-CGCATACAGTCTTRCAGGCT-3′, Reverse: 5′-GTGTGATGTTGAWATGACATGGTC-3′, probe: 5′-FAM-TTAAGATGTGGTGCTTGCATACGTAGAC-lABkFQ-3′) (56).

### Infectious virus titration by plaque assays.

Infected K18-hACE2 mice were euthanized at 2 or 4 dpi The harvested lung and nasal turbinate were homogenized in DMEM with Tissue Lyzer II, followed by centrifugation. To titrate the infectious virus titer, tissue homogenates were 10-fold serially diluted with DMEM and applied to VeroE6-TMPRSS2 cells for 2 hours at 37°C. After inoculation, cells were washed once before overlaying with 1% low-melting agarose containing 1% FBS. Cells were further incubated for 48 hours and fixed with 10% neutral-buffered formalin and visualized with 0.5% crystal violet for plaque formation.

### Immunofluorescence and H&E staining.

After fixing for 24 hours in 10% neutral-buffered formalin, mouse tissues were processed, paraffinized, and sectioned to prepare 4 μm tissue sections on glass slides. After dewaxing, antigen retrieval was performed. SARS-CoV-2 nucleocapsid (N) protein was detected by an in-house rabbit polyclonal anti-SARS-CoV-2 N Ab followed by detection with the Alexa488 goat anti-rabbit Ab (Thermo Fisher Scientific). Nuclei were stained with the DAPI dye (Thermo Fisher Scientific) before the sections were mounted with the Diamond Prolong Antifade mounting buffer (Thermo Fisher Scientific) as we previously described (57). For H&E staining, tissue sections were stained with Gill’s H&E Y (Thermo Fisher Scientific) as we previously described (58). Images were acquired with the Olympus BX53 light microscope.

### Bulk RNA-seq and data analysis.

Total RNA was extracted with the Qiagen RNeasy mini kit (74106). RNA samples which passed quantity control using Agilent 2100 Bioanalyzer were moved forward to paired-end 100 bp sequencing library construction and sequencing on BGISEQ-500. Low quality reads and adaptors were removed from raw sequencing data. Gene expression was estimated by Salmon with mouse reference (genome reference: GRm38; gene annotation source version: GENECODE vM25) (59). The differential expression test was executed between BCG-vaccination and nonvaccination samples belonging to the same tissue and infection state with the help of DESeq2 (60), genes with adjusted *P* values less or equal to 0.05 and fold changes larger or equal to 2 were considered to be significantly differentially expressed. Gene ontology (GO) functional enrichment analysis on significance upregulated genes in BCG-vaccinated BM tissue was carried out in R package clusterProfiler using the default parameter (61). To further investigate the immune-related pathway activation level, we applied GSVA on normalized gene expression (normalized using the vst function in DESeq2 package) with default parameters, and the immune-related pathway gene sets were downloaded from Reactome (version 77) (62). Cell type enrichment was estimated with the help of xCell using the default cell type gene signature in the package (63).

### Flow cytometry.

RBCs in BM cells from mice were lysed using red cell lysis buffer (Thermo Fisher Scientific, 89900) at room temperature for 4 minutes and then washed with PBS (containing 1% BSA) 2 times. For LKS, HSC, and MPP staining, APC mouse lineage antibody cocktail (BD Biosciences, 558074); anti-CD117 (c-kit) rat monoclonal antibody (APC/Cy7) (BioLegend, 105825); Ly-6A/E (Sca-1) monoclonal antibody (D7) (eBioscience, 25-5981-81); PE-Cyanine7 CD150 monoclonal antibody (mShad150), eFluor 450 (eBioscience, 48-1502-80); CD48 monoclonal antibody (HM48-1); PerCP-eFluor 710 (eBioscience, 46-0481-80); PE rat anti–mouse CD135 (BD Biosciences, 561068); CD34 monoclonal antibody (RAM34); and FITC (eBioscience, 11-0341-81) were added to BM cells and were incubated together on ice for 30 minutes. For myeloid cells including monocyte, macrophage, DC, and monocyte staining, APC anti–mouse CD115 (CSF-1R) antibody (BioLegend, 135509); anti–Ly-6C rat monoclonal antibody (Brilliant Violet 605, HK1.4) (BioLegend, 128035); PE/Cy7 anti–mouse Ly-6G Antibody [1A8] (BioLegend, 127617); CD11c armenian hamster anti–mouse Alexa Fluor 700 (Thermo Fisher Scientific, 56-0114-80); anti–mouse CD11b eFluor 450 (eBioscience, 48-0112-80); F4/80 monoclonal antibody (BM8); and Alexa Fluor 488 (BioLegend, 123120) were added to BM cells and were then incubated together on ice for 30 minutes.

### Plasma collection and lipid extraction for metabolomics.

Blood samples from K18-hACE2 mice were drawn from the tail vein on day 14 following BCG vaccination. The plasma was separated by centrifugation at 690*g* for 10 minutes. Metabolite extraction for LC-MS and GC-MS was performed according to a previously described protocol (64). For LC-MS, 50 μL of plasma was first added with 20 μL of ice-cold methanol containing internal standards and butylated hydroxytoluene (BHT). Samples were mixed by a vortex for 5 seconds and kept on ice throughout the extraction procedure. Then, 190 μL of chloroform/methanol (v/v 14:5) was added, followed by mixing with a vortex for 30 seconds twice on ice. The samples were next incubated for 5 minutes at 250*g* at 4°C in an orbital mixer. Next, samples were centrifuged at 1340*g* for 10 minutes at 4°C. The upper phase was dried in a Labconco Centrivap cold trap concentrator for storage at –80°C. Similarly, for GC-MS, 20 μL of plasma was added with 80 μL of chloroform/methanol (v/v 2:1) followed by mixing twice with a vortex for 30 seconds. The samples were incubated for 5 minutes at 250*g* at 4°C in an orbital mixer. After that, the samples were centrifuged at 1340*g* for 10 minutes at 4°C. The upper phase was dried for further analysis.

### (LC-MS–based untargeted metabolomics.

Ultra-performance LC coupled to quadrupole time-of-flight MS (UPLC-Q-TOF-MS) analytical platform (Waters) was used to perform untargeted metabolomics for hydrophilic metabolite characterization (65, 66). The chromatography was performed on a Waters Acquity UPLC BEH Amide column (150 × 2.1 mm; 1.7 μm). The mobile phases and gradient elution were performed according to a previously described protocol with slight modification (67). The mass spectrometer was operated in MS^E^ mode, and the data were acquired in both positive and negative modes. Mass spectral data were acquired over the m/z range of 100–1000. Collision energy was applied at the 20–40 eV range for fragmentation to allow putative identification and structural elucidation of metabolites. Exogenous metabolite standards were applied for sample preparation and LC-MS analysis for monitoring the metabolite coverage and extraction efficiency.

A total of 8 lipid internal standards were applied for sample preparation and LC-MS analysis for monitoring extraction efficiency, including Succinic acid-d6-ISTD, L-Leucine-d10-ISTD, Salicylic acid-d4-ISTD, L-GLUTAMINE-d5-ISTD, Creatine-d3-ISTD, L-arginine-15N2-ISTD, Trimethylamine N-oxide-d9-ISTD, and Butyric acid-d7-ISTD. Commercial standards were used for metabolite identification. All standards were purchased from Cambridge isotope lab or Cayman Chemical.

### GC-MS–based targeted metabolomics.

For polar metabolites, GC-MS/MS chromatogram was acquired in SCAN and MRM mode in an Agilent 7890B GC-Agilent 7010 Triple Quadrapole Mass Spectrometer system. The sample was separated through an Agilent DB-5MS capillary column (30 m × 0.25 mm ID, 0.25 μm film thickness) under constant flow at 1 mL per minute –1. The GC oven program started at 50°C (hold time 1 min) and was increased 10°C per minute –1 to 120°C, then increased 3°C per minute –1 to 150°C, then increased 10°C per minute –1 to 200°C, and finally was increased 30°C per minute –1 to 280°C (hold time 5 min). Inlet temperature and transfer line temperature were 250°C and 280°C, respectively. Characteristic quantifier and qualifier transitions were monitored in MRM mode during the run. Mass spectra from m/z 50–500 were acquired in SCAN mode (68).

### Data processing, statistical analysis, and metabolite identification in untargeted metabolomics.

Untargeted metabolomics study data were processed to a usable data matrix by the MS-DIAL software for further statistical analysis (69). MetaboAnalyst 4.0 (http://www.metaboanalyst.ca) was used for univariate and multivariate analysis. The FDR adjusted *P* value < 0.05 and fold change greater than 1.25 or less than 0.8 were used as the criteria for selecting significant features. In multivariate analysis, partial least squares discriminant analysis (PLS-DA) was applied to identify important variables with discriminative power. The variable importance in projection (VIP), which reflects both the loading weights for each component and the variability of the response explained by this component, was used to select the features. Significant metabolite features were identified by searching accurate MS and MS/MS fragmentation pattern data in the public databases including MassBank of North America (MoNA, http://mona.fiehnlab.ucdavis.edu/) and METLIN database (http://metlin.scripps.edu/). For confirmation of metabolites identity using authentic chemical standards, the MS/MS fragmentation patterns of the chemical standards were compared with those of the candidate lipids measured under the same LC-MS condition. Pathway analysis was performed by Metaboanalyst (70).

### Statistics.

Data visualization and analysis were performed with GraphPad prism 8.0. All statistical details of experiments were specified in the respective figure legends. Statistical comparison between different groups was performed by 1-way ANOVA, 2-way ANOVA, or unpaired 2-tailed Student’s *t* test. Differences were considered statistically significant when * represented *P* < 0.05, ** represented *P* < 0.01, and *** represented *P* < 0.001.

### Study approval.

The use of animals was approved by the Committee on the Use of Live Animals in Teaching and Research (CULATR) of The University of Hong Kong (CULATR 5479-20).

### Data and materials availability.

All data are available in the main text or the supplementary materials. Raw bulk RNA-seq data reported in this article are available in the NCBI Sequence Read Archive with BioProject accession number PRJNA824605.

## Author contributions

BZZ, JDH, and HC designed the study. BZZ, HS, HRG, and BY performed the experiments. BZZ, JCH, BY, MY, JFWC, JDH, and HC analyzed the data. TTTY, YFH, CY, XLW, YH, XL, XH, RL, YMAY, WL, BH, YC, and JPC participated in experiments. XLW, GSL, IFNH, KYY, and JFWC provided key reagents and suggestions. BZZ, HS, JCH, BY, JFWC, JDH, and HC wrote the manuscript. Order of co–first authors was determined based on the timeline of the authors’ contributions.

## Supplementary Material

Supplemental data

## Version 1 04/21/2022

In-Press Preview

## Version 2 06/08/2022

Electronic publication
