# Supplementary material for: Bacillus Calmette-Guérin–induced trained immunity protects against SARS-CoV-2 challenge in K18-hACE2 mice
Source: JCI Insight. 2022 Jun 8;7(11):e157393. doi: 10.1172/jci.insight.157393 (PMC9220951; doi:10.1172/jci.insight.157393)
Supplement: Supplemental data [file jciinsight-7-157393-s199.pdf]

Bacillus Calmette–Guérin-Induced Trained Immunity Protects Against SARS-CoV-2 Challenge in K18-hACE2 Mice

Bao-Zhong Zhang[1,5,†], Huiping Shuai[2,3,4,†], Hua-Rui Gong[5,†], Jing-Chu Hu[1,†], Bingpeng Yan[2,3,4,†], Terrence Tsz-Tai Yuen[2,3,4], Ye-Fan Hu[5,6], Chaemin Yoon[2,3,4], Xiao-Lei Wang[5], Yuxin Hou[2,3,4], Xuansheng Lin[5], Xiner Huang[2,3,4], Renhao Li[5]，Yee Man Au-Yeung[5],Wenjun Li[1], Bingjie Hu[2,3,4], Yue Chai[2,3,4], Ming Yue[5], Jian-Piao Cai[2,3,4], Guang Sheng Ling[5]，Ivan Fan-Ngai Hung[6,7], Kwok-Yung Yuen[2,3,4,7], Jasper Fuk-Woo Chan[2,3,4,7,*], Jian-Dong Huang[1,5,8,*], Hin Chu[2,3,4,7*]

Affiliations:

[1] CAS Key Laboratory of Quantitative Engineering Biology, Shenzhen Institute of Synthetic Biology，Shenzhen Institutes of Advanced Technology, Chinese Academy of Sciences, Shenzhen 518055, China

[2] State Key Laboratory of Emerging Infectious Diseases, Li Ka Shing Faculty of Medicine, The University of Hong Kong, Pokfulam, Hong Kong Special Administrative Region, China

[3] Department of Microbiology, Li Ka Shing Faculty of Medicine, The University of Hong Kong, Pokfulam, Hong Kong Special Administrative Region, China

[4]Centre for Virology, Vaccinology and Therapeutics, Hong Kong Science and Technology Park, Hong Kong Special Administrative Region, China

[5] School of Biomedical Sciences, Li Ka Shing Faculty of Medicine, The University of Hong Kong, Pokfulam, Hong Kong Special Administrative Region, China

[6] Department of Medicine, Li Ka Shing Faculty of Medicine, The University of Hong Kong, Pokfulam, Hong Kong Special Administrative Region, China

[7] Department of Clinical Microbiology and Infection Control, The University of Hong Kong-Shenzhen Hospital, Shenzhen, Guangdong Province, China

[8]Guangdong-Hong Kong Joint Laboratory for RNA Medicine, Sun Yat-Sen University, Guangzhou, China 510120.

† These authors contributed equally to this work.

* Corresponding authors.

Dr. Hin Chu,

Address: 19-044 Block T, Queen Mary Hospital, 102 Pokfulam Road, Department of Microbiology, The University of Hong Kong

Phone：+852-95052096

E-mail: hinchu@hku.hk

Prof. Jian-dong Huang,

Address: L3-72, Laboratory Block, 21 Sassoon Road, School of Biomedical Sciences, The University of Hong Kong

Phone：+852-3917 6810

E-mail: jdhuang@hku.hk

Dr. Jasper Fuk-Woo Chan,

Address: 17th Floor, Block T, Queen Mary Hospital, 102 Pokfulam Road. Department of Clinical Microbiology and Infection Control, The University of Hong Kong-Shenzhen Hospital, Shenzhen, Guangdong Province, China; and State Key Laboratory of Emerging Infectious Diseases, Department of Microbiology, School of Clinical Medicine, The University of Hong Kong

Phone：+852-22552413

E-mail: jfwchan@hku.hk

Supplemental Figures and Figure Legends

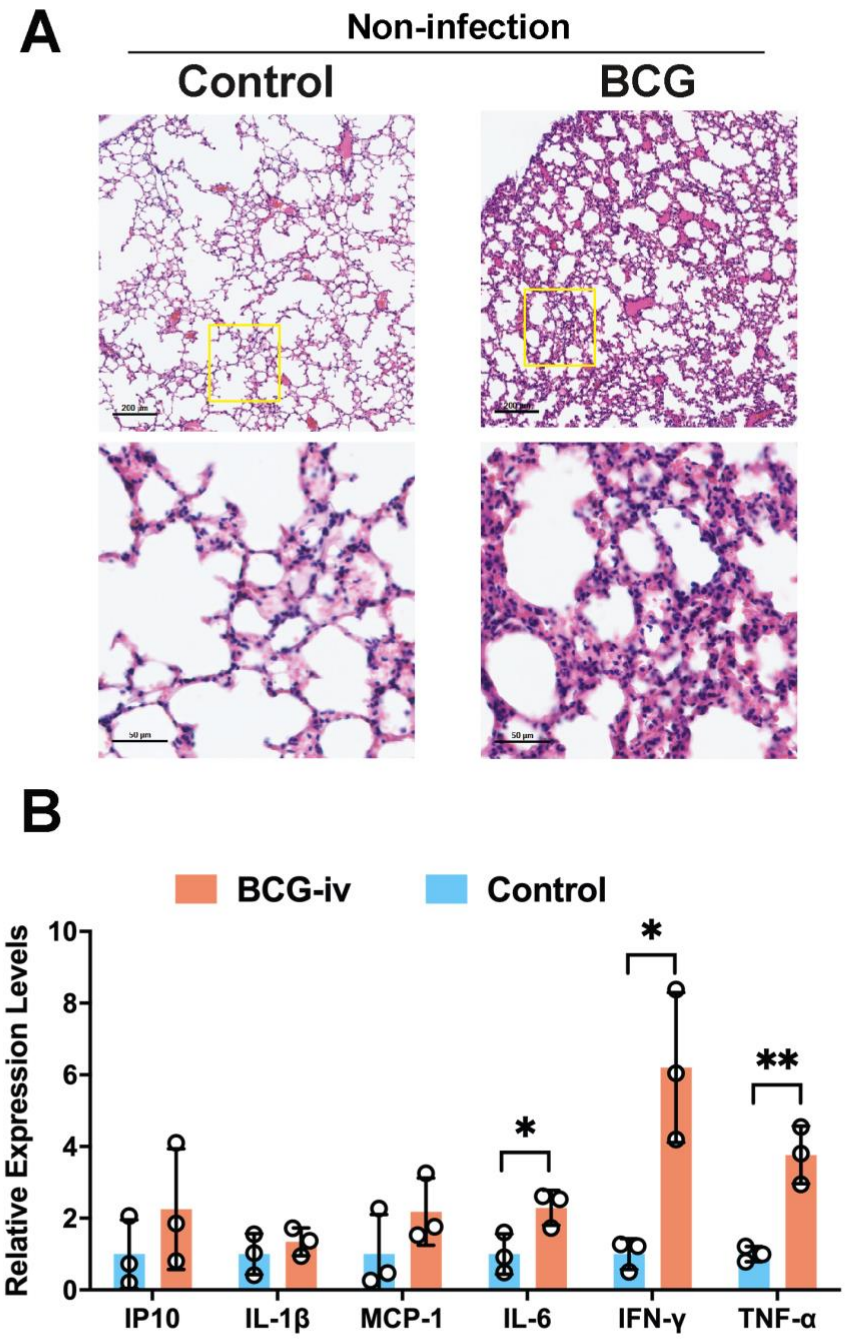

Supplemental Figure 1: (A) Representative images of the H&E-stained lung tissues of Control or BCG-iv at 45 days after initial immunization. Scale bar, 200 μm (top) or 50 μm (bottom). (B) qPCR analysis of IL-6, IP10, IL-1β, TNF-α, IFN-γ, and MCP-1 mRNA expression in lung tissues harvested from K18-hACE2 mice treated with PBS or BCG-iv at 45 days after initial immunization. (n = 3, Student's t test).

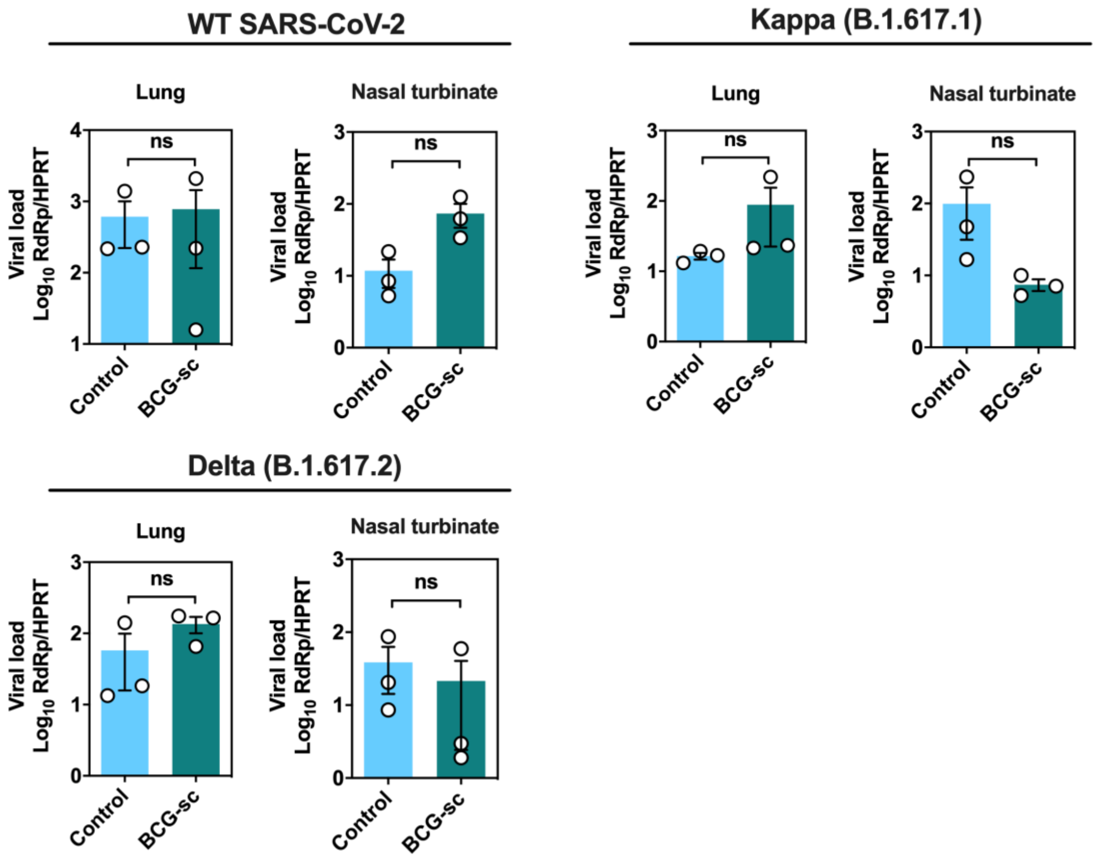

Supplemental Figure 2: After 45 days of subcutaneous BCG (BCG-sc) vaccination, K18-hACE2 mice were intranasally inoculated with 1.25×10^4 plaque-forming units (p.f.u.) of SARS-CoV-2 wild-type or variants. The mice were sacrificed at 2 days after virus challenge for tissue collection. Virus load was determined with qPCR analysis.

Metabolite id	Significant metabolites	Fold change (BCG/control)	P-value	HMDB	KEGG	Identification information	Instrument type
12	Dihydroxyacetone phosphate	2.46	0.0006	HMDB0001473	C00111	standard confirm	GC-MS
7	Aspartic acid	1.92	0.0078	HMDB0000191	C00049	standard confirm	GC-MS
1	Pyruvic acid	1.52	0.0001	HMDB0000243	C00022	standard confirm	GC-MS
8	Phosphoenolpyruvic acid	1.31	0.0013	HMDB0000263	C00074	standard confirm	GC-MS
2	Lactic acid	1.26	0.0004	HMDB0000190	C00186	standard confirm	GC-MS
14	Citric acid	0.72	0.0015	HMDB0000094	C00158	standard confirm	GC-MS
3	3-Hydroxybutyric acid	0.39	0.0062	HMDB0000357	C01089	standard confirm	GC-MS
9	Alpha-ketoglutaric acid	0.34	0.0000	HMDB0000208	C00026	standard confirm	GC-MS
5	Fumaric acid	0.33	0.0000	HMDB0000134	C00122	standard confirm	GC-MS
4	Succinic acid	0.31	0.0000	HMDB0000254	C00042	standard confirm	GC-MS
6	Malic acid	0.23	0.0000	HMDB0000744	C00149	standard confirm	GC-MS
1044	L-Kynurenine	3.16	0.0290	HMDB0000684	C00328	MS2 confirm	LC-MS
144	Uracil	2.87	0.0017	HMDB0000300	C00106	standard confirm	LC-MS
512	Propionylcarnitine	2.40	0.0008	HMDB0000824	C03017	MS2 confirm	LC-MS
55	Cytosine	1.84	0.0000	HMDB0000630	C00380	MS2 confirm	LC-MS
554	2'-Deoxycytidine	1.82	0.0000	HMDB0000014	C00881	MS2 confirm	LC-MS
151	1-Methylnicotinamide	1.78	0.0232	HMDB0000699	C02918	MS2 confirm	LC-MS
599	7,8-Dihydrobiopterin	1.60	0.0404	HMDB0000038	C02953	MS2 confirm	LC-MS
743	D-Mannose	0.66	0.0027	HMDB0000169	C00936	standard confirm	LC-MS
527	5-Hydroxytryptophan	0.65	0.0001	HMDB0000472	C00643	MS2 confirm	LC-MS
130	Serine	0.63	0.0369	HMDB0062263	C00716	standard confirm	LC-MS
355	L-Tyrosine	0.61	0.0032	HMDB0000158	C00082	standard confirm	LC-MS
742	D-Glucose	0.58	0.0050	HMDB0000122	C00031	standard confirm	LC-MS
615	Equol	0.57	0.0282	HMDB0002209	C14131	MS2 confirm	LC-MS
1428	Thymidine	0.52	0.0128	HMDB0000273	C00214	MS2 confirm	LC-MS
1090	Indoxyl sulfate	0.49	0.0105	HMDB0000682	NA	MS2 confirm	LC-MS
420	L-Methionine	0.49	0.0032	HMDB0000696	C00073	standard confirm	LC-MS
401	L-Glutamic acid	0.48	0.0386	HMDB0000148	C00025	standard confirm	LC-MS
278	N-Methylglutamic acid	0.47	0.0000	HMDB0062660	C01046	MS2 confirm	LC-MS
1466	beta-Hydroxymyristic acid	0.46	0.0220	HMDB0010731	NA	MS2 confirm	LC-MS
292	Fluorene	0.45	0.0000	METPA0947	C07715	MS2 confirm	LC-MS
87	Nicotinamide	0.45	0.0467	HMDB0001406	C00153	MS2 confirm	LC-MS
124	3-Hydroxyproline	0.44	0.0002	HMDB0059659	C05147	MS2 confirm	LC-MS
1859	LPC 20:5	0.43	0.0016	HMDB0010397	C04230	MS2 confirm	LC-MS
438	o-Phosphothreonine	0.39	0.0000	HMDB0011185	C12147	MS2 confirm	LC-MS
156	Trigonelline	0.38	0.0000	HMDB0000875	C01004	MS2 confirm	LC-MS
183	L-Threonine	0.35	0.0003	HMDB0000167	C00188	standard confirm	LC-MS
1284	2-Deoxyuridine	0.31	0.0006	HMDB0000012	C00526	MS2 confirm	LC-MS
847	Citric acid	0.31	0.0102	HMDB0000094	C00158	standard confirm	LC-MS
131	3-Ureidopropionic acid	0.27	0.0017	HMDB0000026	C02642	MS2 confirm	LC-MS
396	L-Lysine	0.25	0.0002	HMDB0000182	C00047	standard confirm	LC-MS
192	Indole-3-carboxaldehyde	0.24	0.0001	HMDB0000195	C00294	MS2 confirm	LC-MS
469	L-Tryptophan	0.23	0.0000	HMDB0000929	C00078	standard confirm	LC-MS
677	L-Arginine	0.19	0.0000	HMDB0000517	C00062	standard confirm	LC-MS
388	Alpha-ketoglutaric acid	0.18	0.0298	HMDB0000208	C00026	standard confirm	LC-MS
294	Malic acid	0.16	0.0001	HMDB0031518	C00149	standard confirm	LC-MS
149	Hypoxanthine	0.01	0.0173	HMDB0000157	C00262	MS2 confirm	LC-MS
440	3-Hydroxyphenylacetic acid	0.00	0.0374	HMDB0000440	C05593	MS2 confirm	LC-MS

Supplemental Table 1. The 45 plasma metabolites that were significantly different between BCG training and control-treated mouses

Primer Name	5'-3'
IP-10	(F) ATGACGGGCCAGTGAGAATG
	(R) GAGGCTCTCTGCTGTCCATC
IL-1β	(F) GCCTTGGGCCTCAAAGGAAAGAATC
	(R) GGAAGACACAGATTCCATGGTGAAG
IL-6	(F) TGGAGTCACAGAAGGAGTGGCTAAG
	(R) TCTGACCACAGTGAGGAATGTCCAC
MCP-1	(F) GGCTCAGCCAGATGCAGTTAA
	(R) CCTACTCATTGGGATCATCTTGCT
TNF-a	(F) ATAGCTCCCAGAAAAGCAAGC
	(R) CACCCCGAAGTTCAGTAGACA
IFN-γ	(F) AAGCGTCATTGAATCACACC
	(R) CGAATCAGCAGCGACTCCTT

Supplemental Table 2. Sequences of primers and probes for real time RT-qPCR detection of mRNA gene expression of host cytokines
